# Mortality, Cardiovascular Disease, and Their Associations With Risk Factors in Southeast Asia: A PURE Substudy

**DOI:** 10.1016/j.jacasi.2024.05.008

**Published:** 2024-07-30

**Authors:** Aditya K. Khetan, Lia M. Palileo-Villanueva, Nafiza Mat-Nasir, Rosnah Ismail, Antonio Miguel Dans, Marc Evans M. Abat, Shofiqul Islam, Philip Joseph, Darryl P. Leong, Koon K. Teo, Sumathy Rangarajan, Salim Yusuf

**Affiliations:** a Population Health Research Institute, McMaster University and Hamilton Health Sciences, Hamilton, Ontario, Canada; b UP College of Medicine, University of the Philippines, Manila, Philippines; c Department of Primary Care Medicine, Faculty of Medicine, MARA Technological University, Sungai Buloh Campus, Selangor, Malaysia; d Faculty of Medicine, Kebangsaan University, Selangor, Malaysia; e Department of Medicine, University of Philippines, Manila, Philippines; f Department of Medicine, Philippine General Hospital, Manila, Philippines

**Keywords:** cardiovascular disease, modifiable risk factors, mortality, Southeast Asia

## Abstract

**Background:**

The drivers of cardiovascular disease (CVD) and all-cause mortality may differ around the world. Regional-level prospective data can help guide policies to reduce CVD and all-cause mortality.

**Objectives:**

This study examined the incidence of CVD and mortality in Malaysia and the Philippines and estimated the population-level risks attributable to common risk factors for each outcome.

**Methods:**

This prospective cohort study included 20,272 participants from Malaysia and the Philippines. The mean follow-up was 8.2 years. The incidences of CVD and mortality rates were calculated for the overall cohort and in key subgroups. For each outcome, population-attributable fractions (PAFs) were calculated to compare risks associated with 12 modifiable risk factors.

**Results:**

The mean age of the cohort was 51.8 years (59% women). Leading causes of mortality were CVD (37.9%) and cancer (12.4%). The incidence of CVD (per 1,000 person-years) was higher in the Philippines (11.0) than Malaysia (8.3), and CVD contributed to a higher proportion of deaths in the Philippines (58% vs 36%). By contrast, all-cause mortality rates were higher in Malaysia (14.1) than in the Philippines (10.9). Approximately 78% of the PAF for CVD and 68% of the PAF for all-cause mortality were attributable to 12 modifiable risk factors. For CVD, the largest PAF was from hypertension (24.2%), whereas for all-cause mortality, the largest PAF was from low education (18.4%).

**Conclusions:**

CVD and cancer account for one-half of adult mortality in Malaysia and the Philippines. Hypertension was the largest population driver of CVD, whereas low education was associated with the largest burden of overall mortality.

The burden of cardiovascular disease (CVD), all-cause mortality, and their determinants may differ in various countries. Understanding these differences is essential in developing context-specific health policies. Health policies aimed at reducing CVD and all-cause mortality can be better informed from contemporary data on these 2 vital outcomes, to understand their drivers and variation by sex. This can help guide strategies for prevention and treatment within these countries. In particular, there is a lack of prospective data on CVD and all-cause mortality in many countries in Southeast Asia, with existing data largely modeled estimates (eg, from the Global Burden of Disease).^1^ Prospective studies can document the incidence rates of CVD and all-cause mortality and clarify the contribution of risk factors. A standardized cohort of participants from countries within Southeast Asia can provide necessary information on whether the rates of CVD and all-cause mortality vary, as well as region-specific data on their determinants. Southeast Asian countries are often analyzed as a homogenous group, even though there are significant differences among many of these countries. For instance, there is a wide variation in religion or culture and government within the region that may affect the risk factor profile and outcomes in an individual country. Malaysia is a federal constitutional monarchy, with most of its citizens being Sunni Muslims. The Philippines, conversely, is a presidential republic, with 90% of its citizens practicing Christianity.^2^

The PURE (Prospective Urban Rural Epidemiology) study is a large, community-based study that is evaluating the rates and determinants of CVD and mortality from 27 high-, middle-, and low-income countries. Using standardized methods, we have collected data on a broad range of health determinants and clinical outcomes in participants from 74 communities in Malaysia and the Philippines (2 of the larger countries in the region) during a mean follow-up period of 8.2 years. In this cohort, we aimed to characterize whether CVD incidence and mortality rates varied in key subgroups, by sex and between the 2 countries, and to quantify the contributions of the common modifiable risk factors to these outcomes.

## Methods

### Study design and participants

PURE is a prospective cohort study of community-based adults aged 35 to 70 years from high-, middle-, and low-income countries across 6 geographic regions: Asia, Africa, Europe, South America, North America, and the Middle East. The study design has been published previously.^3^ The present study focuses on participants from Malaysia and the Philippines, who were recruited from 28 rural and 46 urban communities at 3 collaborating centres. Using prespecified guidelines, a sample of households from each community with at least 1 member aged between 35 and 70 years (and intending to reside at the current home for at least 4 years) was invited to participate in the study. PURE is broadly reflective of its participating countries with regard to population demographics and mortality rates.^4^ Eligible persons provided written informed consent, and the study was approved by local ethics committees at each site. A total of 20,272 individuals with at least 1 follow-up visit, enrolled since 2007, were included in the cohort. Follow-up events recorded until April 2023 were included in the analysis.

### Data collection

Baseline information was collected using standardized questionnaires and physical measurements. On the basis of their established association with both CVD and mortality shown in our global data, 12 risk factors collected at baseline were evaluated in this analysis.^5^ The definitions of the risk factors and their thresholds for calculation of population-attributable fractions (PAFs) are described in detail in Supplemental Methods and Supplemental Table 1 and are briefly summarized here. These risk factors were metabolic risk factors (hypertension, diabetes, elevated non–high density lipoprotein [HDL] cholesterol, triglycerides, and abdominal obesity), behavioral risk factors (tobacco use, alcohol use, diet quality, and physical activity), low education level, household air pollution, low strength (on the basis of grip strength), and depression.

Hypertension was defined as a self-reported history of hypertension, a baseline systolic blood pressure ≥140 mm Hg, a diastolic blood pressure ≥90 mm Hg, or use of blood pressure–lowering medications. Diabetes was defined as a baseline fasting glucose level ≥126 mg/dL, a self-reported history of diabetes, or treatment with a glucose-lowering agent. Waist and hip circumferences were measured using a standard protocol, and abdominal obesity was defined as a waist-to-hip ratio >0.9 in men and >0.85 in women. Smoking and alcohol use were collected and quantified by self-report. We used a composite diet score for overall diet quality, which has been replicated in 5 independent cohorts.^6^ Physical activity was measured using the International Physical Activity Questionnaire.^7^ Education level was collected as part of the baseline assessment and was chosen as the primary socioeconomic variable of interest because education was found to be a stronger socioeconomic predictor of CVD and mortality than wealth or income in a previous PURE analysis.^8^ Grip strength was measured by a Jamar dynamometer. Household air pollution was collected at the household level and was defined as the use of kerosene or solid fuels as the primary fuel for cooking. Depression was defined as a score of at least 5 on an 8-symptom score of the Composite International Diagnostic Interview.^9^ Fasting non-HDL cholesterol was chosen as the primary lipid value of interest because it had the strongest association with CVD in our global risk factor paper. Categories of risk for each risk factor were based on those used in the report on PURE global risk factors.^5^

This approach allows for consistent thresholds of risk to compare across different regions. Wealth, calculated at the household level, was defined by an index on the basis of ownership of assets and housing characteristics.^8^ This index has been previously validated in low-, middle- and high-income countries and documented to be a robust measure of wealth, consistent with measures of income and expenditure.^10^ Participants were categorized into 20 equal groups, on the basis of their wealth percentile, with higher categories denoting greater wealth relative to others from the global cohort in lower-wealth categories.

### Follow-up and outcomes

During follow-up, clinical outcomes were recorded at each study visit by using standardized case report forms. A CVD event in follow-up was defined as the composite of cardiovascular mortality, myocardial infarction, stroke, or heart failure. Outcome events were adjudicated centrally by trained physicians using standardized procedures and definitions. Cause-specific mortality was classified using supporting documentation, which included a standardized verbal autopsy form if the cause of mortality was initially classified as unknown. Follow-up in the cohort was scheduled yearly by telephone and every 3 years in the home or health facility. Mortality events were also routinely reviewed centrally by a trained nosologist and classified using all available data. The cause of mortality was classified by the International Classification of Diseases-10th revision (ICD-10) code, or an alternative study code if not available through the ICD-10 framework (Supplemental Methods).

### Statistical analysis

Baseline characteristics are described in the overall study group, by country, and by sex. Age- and sex-standardized incidence rates for CVD and for all-cause mortality were calculated per 1,000 person-years overall, by country, and by sex. Direct standardization to the overall Southeast Asian cohort was applied to estimates in each subgroup. Leading causes of mortality are presented for the overall cohort. Associations among individual risk factors, CVD, and mortality were examined in those participants without a previous history of CVD, by using a multivariable Cox shared frailty model, which was mutually adjusted for each risk factor in addition to age, sex, and urban or rural location. Each community was included as a random intercept in the model, and the probability distribution was gamma. For some risk factors, data were missing because some questions were not implemented at the initiation of the study, or fasting lipids were not collected in a subset of participants. To address this issue, we used multiple imputation for missing data for each risk factor planned for analysis. We used the multiple imputation procedure in SAS software version 9.4 (SAS Institute, Inc), under the assumption that the missing data pattern is random. In particular, the regression-based fully conditional specification method was used in this imputation.^11^ Five imputed data sets were created for each missing value. Although we did not perform any formal test to check for a missing data mechanism, we do not have adequate information to believe that the missing data pattern is not at random. Estimates are based on the multiple imputation data set and are presented as HRs with 95% CIs.

The PAF estimated the contribution of a risk factor to the development of CVD or to all-cause mortality. PAFs for each individual or groups of risk factors were estimated using the average PAF approach by Eide and Gefeller^12^ and the averisk package in R software (R Foundation) developed by Ferguson et al.^13^ Each risk factor of interest was dichotomously categorized based on a prespecified threshold level of risk on the basis of the global PURE report (Supplemental Methods, Supplemental Tables 1 and 2).^5^ This approach allows for comparisons with the global PURE data and across different geographic regions. Average PAF estimates were derived using a mutually adjusted model accounting for all candidate risk factors, age, sex and urban or rural location; where each risk factor was added sequentially to the model in all possible permutations and then the average PAF from these permutations was calculated. In this approach, cumulative PAF estimates for groups of risk factors are additive and cannot exceed 100%. In general, the average PAF method results in smaller PAF estimates for individual risk factors compared with other approaches. For groups of risk factors, each risk factor’s contribution was truncated at a lower limit of 0 because this is the lowest theoretical level of risk that can be attributable to a risk factor. PAFs were not calculated at the country level given the limited numbers of outcome events to perform these estimates after stratifying by country. Analyses were conducted using Stata (StataCorp) and R software.

## Results

### Baseline characteristics of the study group

We enrolled 15,761 participants from Malaysia and 4,511 participants from the Philippines. Baseline characteristics overall, by sex, and by country are summarized in Table 1. The mean age was 51.8 ± 9.8 years and 59% of participants were women. A total of 56% of participants were from rural areas. Of metabolic risk factors, 48% had hypertension, whereas 15% had diabetes.

**Table 1:** Baseline Characteristics of the Study Participants Overall, by Sex, and Country

	Country		Sex	
	Malaysia, (n = 15,761)	Philippines, (n = 4,511)	Women, (n = 12,044)	Men, (n = 8,228)
Mean age, y	51.5 ± 9.9	52.6 ± 9.7	51.2 ± 9.7	52.7 ± 9.9
Body mass index, kg/m^2^	26.5 ± 5.0	24.1 ± 4.3	26.0 ± 5.1	25.5 ± 4.6
Waist to hip ratio	0.874 ± 0.082	0.894 ± 0.073	0.859 ± 0.080	0.91 2 ± 0.068
WHR men >0.9 or women >0.85	6,312 (50.6)	2,951 (67.3)	5,374 (52.7)	3,889 (58.4)
Global wealth index	12.9 ± 4.3	6.8 ± 4.5	11.1 ± 5.2	12.0 ± 4.9
PURE diet score	3.0 ± 1.4	2.8 ± 0.9	2.9 ± 1.3	2.9 ± 1.3
Location				
Urban	6,932 (44.0)	2,085 (46.2)	5,259 (43.7)	3,758 (45.7)
Rural	8,829 (56.0)	2,426 (53.8)	6,785 (56.3)	4,470 (54.3)
Education level				
Presecondary school	6,690 (42.5)	958 (21.3)	4,732 (39.3)	2,916 (35.5)
Secondary school	6,947 (44.1)	2,197 (48.8)	5,335 (44.3)	3,809 (46.3)
Postsecondary school	2,113 (13.4)	1,352 (30.0)	1,968 (16.4)	1,497 (18.2)
Physical activity level				
Low	5,005 (34.5)	119 (2.8)	2,691 (23.8)	2,433 (32.4)
Moderate	4,979 (34.3)	1,265 (29.3)	4,010 (35.4)	2,234 (29.8)
High	4514 (31.1)	2,938 (68.0)	4,613 (40.8)	2,839 (37.8)
Tobacco use				
Former	1,239 (8.0)	571 (13.0)	281 (2.4)	1,529 (18.9)
Current	2,369 (15.2)	655 (14.9)	361 (3.0)	2,663 (32.8)
Never	11,984 (76.9)	3,179 (72.2)	11,246 (94.6)	3,917 (48.3)
Alcohol use				
Former	307 (2.0)	485 (11.1)	327 (2.8)	465 (5.7)
Current	300 (1.9)	1852 (42.4)	1118 (9.5)	1034 (12.8)
Never	15,046 (96.1)	2035 (46.6)	10,460 (87.9)	6621 (81.5)
Risk factors				
Hypertension	5,483 (46.5)	2,320 (52.7)	4,641 (47.1)	3,162 (50.0)
Diabetes	2,418 (15.3)	621 (13.8)	1,742 (14.5)	1,297 (15.8)
Total cholesterol, mmol/L	5.5 ± 1.2	5.5 ± 1.2	5.6 ± 1.2	5.4 ± 1.2
LDL cholesterol, mmol/L	3.5 ± 1.1	3.5 ± 1.0	3.6 ± 1.0	3.4 ± 1.0
Non-HDL cholesterol, mmol/L	4.4 ± 1.2	4.2 ± 1.1	4.3 ± 1.2	4.3 ± 1.2
HDL cholesterol, mmol/L	1.16 ± 0.3	1.29 ± 0.3	1.28 ± 0.3	1.10 ± 0.3
Triglycerides, mmol/L	1.80 ± 1.1	1.66 ± 1.2	1.63 ± 1.1	1.92 ± 1.2
Use of solid fuels	238 (1.5)	2,072 (46.0)	1,638 (13.6)	672 (8.2)
Depression	744 (4.8)	206 (4.7)	656 (5.5)	294 (3.6)
Grip strength, kg	26.1 ± 9.9	25.2 ± 8.2	20.9 ± 5.9	33.8 ± 8.7

Values are mean ± SD or n (%).

HDL = high-density lipoprotein; LDL = low-density lipoprotein; PURE = Prospective Urban Rural Epidemiology; WHR = waist to hip ratio.

The mean global wealth index of participants from Malaysia was 12.9 ± 4.3, whereas that of participants from the Philippines was much lower, at 6.8± 4.5. The opposite trend was seen for education, with 13% of participants from Malaysia with a postsecondary education, whereas 30% of participants from the Philippines had a postsecondary education. Although tobacco use was similar in the 2 countries (15% current users), reported alcohol use was higher in the Philippines (42%) than in Malaysia (2%). Participants from the Philippines also had higher levels of physical activity. The PURE diet score was lower (suggesting a lower-quality diet) in the Philippines (2.8 ± 0.9), compared with Malaysia (3.0 ± 1.4), and there was also greater use of solid fuels for cooking in the Philippines.

### Mortality

**Figure 1:**
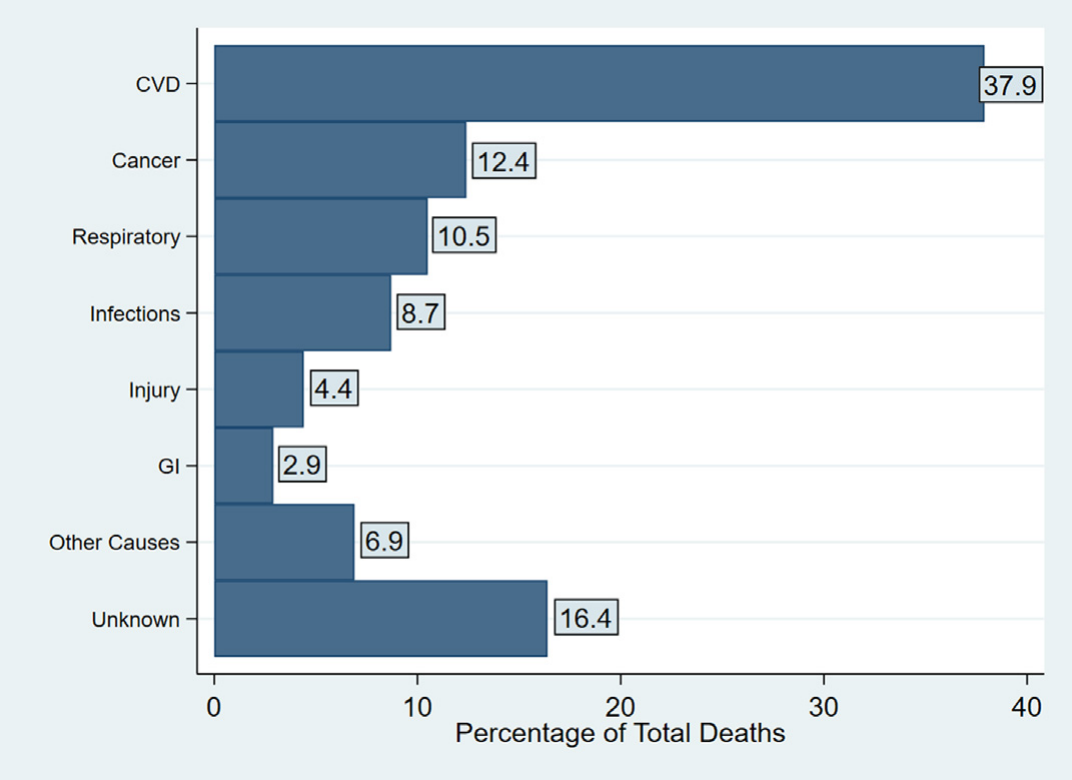
Major Causes of Mortality in the Southeast Asia Cohort The leading causes of death in the Southeast Asia cohort are shown in descending frequency. CVD = cardiovascular disease; GI = gastrointestinal.

A total of 2,226 (11%) members of the cohort died during a follow-up period of a mean of 8.2 ± 4.0 years. The mortality rate was 13.7 (95% CI: 13.1-14.2) per 1,000 person-years. The most common cause of mortality was CVD (38% of mortality), followed by cancer (12%), respiratory diseases (10.5%), infection (8.7%), and injury (4.4%) (Figure 1). Mortality rates were higher in men (18.2 [95% CI: 17.2-19.1] per 1,000 person-years) compared with women (10.0 [95% CI: 9.4-10.6] per 1,000 person-years) (Table 2). Malaysia had a higher mortality rate (14.1 [95% CI: 13.5-14.7] per 1,000 person-years) compared with the Philippines (10.9 [95% CI: 9.3-12.4] per 1,000 person-years). A higher proportion of mortality events in the Philippines was related to CVD (58%, compared with 36% in Malaysia) (Supplemental Methods, Supplemental Table 3).

**Table 2:** Age- and Sex-Standardized Incidence of All-Cause Mortality and Cardiovascular Disease

	Overall	Malaysia	Philippines	Women	Men	Malaysia		Philippines	
						Women	Men	Women	Men
All-cause mortality	13.7 (13.1-14.2) N = 2,262	14.1 (13.5-14.7) n = 2,049	10.9 (9.3-12.4) n = 213	10.0 (9.4-10.6) n = 936	18.2 (17.2-19.1) n = 1,326	10.2 (9.5-10.9) n = 837	19.3 (18.3-20.4) n = 1,212	6.2 (5.1-7.7) n = 99	17.5 (14.2-21.4) n = 114
Major cardiovascular events	8.4 (8.0-8.9) N = 1,390	8.3 (7.8-8.7) n = 1,181	11.0 (9.4-12.6) n = 209	6.0 (5.5-6.5) n = 557	11.9 (11.1-12.7) n = 833	5.5 (5.0-6.1) n = 454	11.7 (10.9-12.6) n = 727	5.9 (4.8-7.4) n = 103	14.5 (11.6-18.2) n = 106
Myocardial infarction	3.7 (3.4-4.0) N = 613	3.6 (3.3-3.9) n = 528	4.4 (3.4-5.4) n = 85	2.1 (1.8-2.4) n = 196	5.9 (5.4- 6.5) n = 417	1.9 (1.6-2.3) n = 158	6.0 (5.4-6.6) n = 370	2.4 (1.7-3.3) n = 38	6.5 (4.6-9.1) n = 47
Stroke	2.8 (2.5-3.1) N = 458	2.4 (2.2-2.7) n = 351	5.7 (4.5-6.8) n = 107	2.3 (2.0-2.7) n = 220	3.3 (2.9-3.7) n = 238	2.0 (1.7-2.3) n = 162	3.0 (2.6-3.5) n = 189	3.4 (2.5-4.5) n = 58	6.5 (4.7-9.1) n = 49
Heart failure	1.0 (0.8-1.1) N = 197	1.2 (1.0-1.4) n = 175	1.1 (0.6-1.6) n = 22	0.8 (0.6-1.0) n = 75	1.7 (1.4-2.0) n = 122	0.7 (0.5-0.9) n = 63	1.5 (1.2-1.8) n = 112	0.2 (0.1-0.7) n = 8	1.0 (0.4-2.3) n = 14

Values are incidence rates (95% CI). Incidence rates are based on 1,000 person-years of follow-up. The number of events for each outcome and category is shown as n. Major cardiovascular events include cardiovascular mortality, myocardial infarction, stroke, or heart failure.

### CVD events

A total of 1,390 CVD events occurred in follow-up, including 613 myocardial infarctions and 458 strokes. The incidence of CVD was 8.4 (95% CI: 8.0-8.9) per 1,000 person-years. The incidence of a CVD event was higher in men (11.9 [95% CI: 11.1-12.7] per 1,000 person-years) compared with women (6.0 [95% CI: 5.5-6.5] per 1,000 person-years). The Philippines had a higher incidence of CVD (11.0 [95% CI: 9.4-12.6] per 1,000 person-years) than Malaysia (8.3 [95% CI: 7.8-8.7] per 1,000 person-years).

The incidence of myocardial infarction was 3.7 (95% CI: 3.4-4.0) per 1,000 person-years. In Malaysia, the incidence of myocardial infarction was 3.6 (95% CI: 3.3-3.9) per 1,000 person-years, whereas it was 4.4 (95% CI: 3.4-5.4) per 1,000 person-years in the Philippines. For stroke, the incidence in the cohort was 2.8 (95% CI: 2.5-3.1) per 1,000 person-years. The Philippines had a higher incidence of stroke (5.7 [95% CI: 4.5-6.8] per 1,000 person-years) than Malaysia (2.4 [95% CI: 2.2-2.7] per 1,000 person-years).

### Associations among modifiable risk factors, CVD, and mortality

A total of 19,554 participants did not have a previous CVD event. Of the risk factors examined for CVD (Table 3), strong associations (ie, HR: ≥1.5) were observed for diabetes (HR: 1.86; 95% CI: 1.63-2.12), hypertension (HR: 1.66; 95% CI: 1.46-1.89), and low education (HR: 1.74; 95% CI: 1.36-2.24). Smaller associations were seen for former and current smoking, abdominal obesity, and elevated triglycerides. Estimates for elevated non-HDL cholesterol, physical inactivity, poor-quality diet, low grip strength, and depression were directionally consistent with a higher CVD risk, but the CIs were wide.

**Table 3:** Associations Among Modifiable Risk Factors, All-Cause Mortality, and CVD

Exposure	All-Cause Mortality HR (95% CI)	CVD HR (95% CI)
Number of events	2,262	1,390
Education		
Postsecondary school	1.00 (reference)	1.00 (reference)
Secondary school	1.65 (1.33-2.03)	1.52 (1.19-1.93)
Presecondary school	2.21 (1.79-2.74)	1.74 (1.36-2.24)
Tobacco use		
Never	1.00 (reference)	1.00 (reference)
Former	1.24 (1.07-1.43)	1.25 (1.04-1.51)
Current	1.43 (1.26-1.61)	1.49 (1.27-1.75)
PURE diet score		
>4	1.00 (reference)	1.00 (reference)
2-4	0.89 (0.77-1.02)	1.06 (0.88-1.28)
≤2	0.94 (0.82-1.08)	1.09 (0.90-1.32)
Physical activity		
High activity	1.00 (reference)	1.00 (reference)
Moderate activity	1.09 (0.98-1.23)	1.05 (0.91-1.21)
Low activity	1.23 (1.10-1.38)	1.08 (0.93-1.25)
Non HDL cholesterol		
Non-HDL < 3.2 mmol/L	1.00 (reference)	1.00 (reference)
Non-HDL 3.2–4.0 mmol/L	0.90 (0.79-1.02)	1.01 (0.85-1.19)
Non-HDL > 4.0 mmol/L	0.90 (0.80-1.02)	1.03 (0.87-1.21)
Triglycerides		
Triglycerides <1.7 mmol/L	1.00 (reference)	1.00 (reference)
Triglycerides 1.7-4.0 mmol/L	1.10 (1.00-1.21)	1.06 (0.93-1.20)
Triglycerides >4.0 mmol/L	1.19 (0.98-1.45)	1.13 (0.88-1.46)
Grip strength		
Quintile 5	1.00 (reference)	1.00 (reference)
Quintile 4	1.06 (0.92-1.22)	1.05 (0.88-1.26)
Quintile 3	1.17 (1.01-1.37)	1.07 (0.88-1.30)
Quintile 2	1.29 (1.10-1.51)	1.02 (0.83-1.27)
Quintile 1	1.34 (1.14-1.57)	1.13 (0.92-1.40)
Hypertension	1.24 (1.13-1.37)	1.66 (1.46-1.89)
Diabetes	1.93 (1.75-2.14)	1.86 (1.63-2.12)
Abdominal Obesity	1.17 (1.06-1.29)	1.35 (1.18-1.53)
Depression	1.33 (1.07-1.65)	1.04 (0.77-1.42)

For all-cause mortality, strong associations were observed for low education (HR: 2.21; 95% CI: 1.79-2.74) and diabetes (HR: 1.93; 95% CI: 1.75-2.14). Smaller associations were seen for current and former smoking, physical inactivity, low grip strength, hypertension, abdominal obesity, elevated triglycerides, and depression. Elevated non-HDL cholesterol and poor-quality diet were not associated with all-cause mortality.

### PAFs for CVD and for all-cause mortality

**Figure 2:**
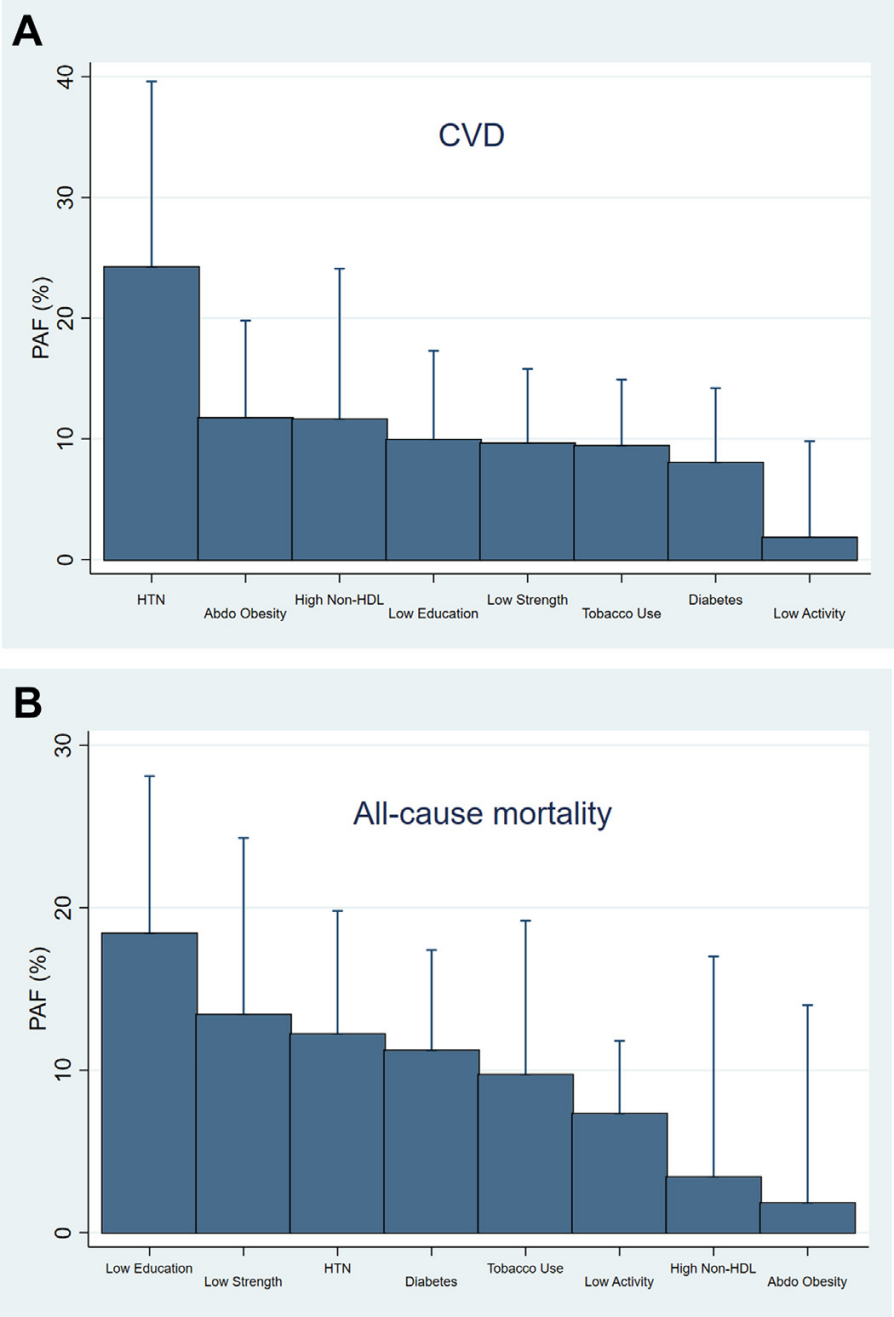
PAF for Cardiovascular Events and All-Cause Mortality (A) Cardiovascular disease (CVD) events. (B) All-cause mortality. The population-attributable fraction (PAF) was mutually adjusted for 12 modifiable risk factors in addition to age, sex, and urban-rural location. Estimates for individual risk factors were truncated at a lower limit of 0 because this is the lowest threshold to demarcate a relationship with increased risk. Abdo = abdominal; HDL = high-density lipoprotein; HTN = hypertension.

Approximately 61% of the PAF for CVD was related to modifiable risk factors. As a cluster, metabolic risk factors (diabetes, hypertension, non-HDL cholesterol, and abdominal obesity) contributed to approximately 56% of the PAF for CVD. Figure 2A summarizes the PAFs for CVD related to individual risk factors. The largest PAFs for CVD were attributable to hypertension (24.2%), abdominal obesity (11.7%), high non-HDL cholesterol (11.6%), low education (9.9%), low strength (9.6%), tobacco use (9.4%), and diabetes (8%).

Approximately 41% of the PAF for all-cause mortality was related to modifiable risk factors. As a cluster, metabolic risk factors (diabetes, hypertension, non-HDL cholesterol, and abdominal obesity) contributed to approximately 29% of the PAF for all-cause mortality. Figure 2B summarizes the PAFs for all-cause mortality related to individual risk factors. The largest PAFs for all-cause mortality were attributable to low education (18.4%), low strength (13.4%), hypertension (12.2%), diabetes (11.2%), tobacco use (9.7%), and low activity (7.3%).

## Discussion

We report the rates of all-cause mortality and CVD and describe their characteristics and risk factors in 2 countries in Southeast Asia. More than 50% of mortality in the cohort resulted from CVD and cancer, with CVD causing 3 times more mortality events than cancer. This finding emphasizes the dominant role of noncommunicable diseases in driving adult mortality in the region, among both women and men.

We found significant differences between Malaysia and the Philippines. CVD caused a staggering 58% of mortality in the Philippines (with particularly high rates of stroke), whereas CVD was the cause of only 36% of mortality in Malaysia. Although the burden of CVD was higher in the Philippines, the rates of all-cause mortality were higher in Malaysia, largely driven by higher rates of infections, injury, and respiratory causes in Malaysia. However, CVD and cancer together caused 50% or more mortality in both countries. Our estimates of causes of mortality are similar to estimates from vital statistics in Malaysia and the Philippines.^1^^,^^14^ There are important differences in risk factors between the 2 countries that could account for some of the differences in mortality patterns. For instance, Filipino households had higher rates of abdominal obesity and solid fuel use compared with Malaysian households, and this difference could lead to increased CVD mortality. Conversely, the higher all-cause mortality rates in Malaysian participants may be driven by lower educational attainment in participants from Malaysia compared with Filipino participants. Education has been shown to have a strong causal effect on mortality, and it was the largest risk factor for mortality (18.4% of the PAF) in our analysis.^15^ The benefits of education likely accrue through various pathways, ranging from economic and psychosocial to improved health behaviors.^16^ Improved health behaviors may account for up to one-third of the effect of education on health.^17^ Improved nutrition and cognitive and noncognitive skill development are other possible pathways mediating the effect of education on health. However, the effects of education on health vary substantially across time and geographies, and much work remains to be done in understanding the precise mechanisms by which education affects health outcomes.^18^ In addition, there is likely some residual confounding in the estimate of education’s effect on mortality, especially from difficult to measure early life deprivations such as stunting and childhood disease burden.^19^ It is notable that Malaysian participants had higher rates of all-cause mortality despite being wealthier than participants from the Philippines. We have previously shown that education is a stronger marker for mortality than wealth, a finding underscoring the importance of education in population health promotion vs policies focused solely on wealth creation.^8^

We found that compared with women, men had nearly twice the rate of all-cause mortality and CVD events. Although higher rates of mortality and CVD events in men are well known, the gap between men and women in our study exceeded the gender gap described in other regions previously (South Asia, China, or South America).^20^, ^21^, ^22^ The exceptionally low rates of current smoking among women in our sample (3%, compared with 33% in men) and the high educational attainment among women may explain some of this gap.^23^

Hypertension was the leading risk factor for CVD (24.2% of PAF). Other metabolic risk factors (diabetes, non-HDL cholesterol, and abdominal obesity) collectively contributed to 31.3% of the PAF for CVD. These 4 risk factors accounted for >50% of the PAF for CVD. Tobacco was ranked sixth as a risk factor for CVD (9.4% of PAF), with hypertension contributing to more than twice the PAF for CVD compared with tobacco. In addition, hypertension had a higher contribution to PAF for mortality than tobacco (12.2% of PAF vs 9.7%). Compared with diabetes, hypertension has a lower HR for all-cause mortality and CVD. However, the much higher prevalence of hypertension (47%, vs 14% for diabetes) leads to a larger PAF and populationwide impact of hypertension. Together, these findings underscore the importance of prevention and better management of hypertension as being central in efforts to decrease the burden of CVD and mortality in Malaysia and the Philippines. A model of care involving nonphysician health workers, primary care physicians, family, and the provision of free medications for hypertension previously showed that it led to 65% of patients attaining control of blood pressure (<140/90 mm Hg) in Malaysia.^24^

Hypertension is the leading contributor to PAF for CVD, a finding that was consistent with our global analysis and with other global data sources.^25^ Similarly, low education as the leading contributor to PAF for mortality was also consistent with our global analysis.^5^ In contrast, tobacco had a lower contribution to both CVD and mortality compared with our global analysis. A total of 15.1% of our cohort were current users of tobacco, significantly lower than rates in South Asia (23.5%), China (22.6%), and South America (20.6%).^20^^,^^21^ Despite these findings, it is crucial that Malaysia and the Philippines continue to also focus on preventing initiation of tobacco use, an approach that will have large benefits in preventing CVD and all-cause mortality.

### Study limitations

Our findings may not be generalizable to other countries in Southeast Asia that are not included in our study. However, these findings emphasize that data from 1 country cannot be applied to another country in the region, and therefore country-specific data are highly desirable. Within Malaysia and the Philippines, our study included 74 communities, but this sample may still not reflect the health profiles in each of these countries, particularly for the Philippines. In the Philippines, the sampled communities were all from Luzon Island (Manila, Quezon, and Rizal provinces). Therefore, our findings may not be generalizable to the island groups of Visayas and Mindanao in the Philippines.^26^ Within our Philippine sample, the prevalence of low education (and other socioeconomic disadvantages) was lower than other areas of the country, and this may explain some of the disparity in educational status between the Philippines and Malaysia in our sample. PAF risk estimates reflect the prevalence of risk factors in our sample, which is predominantly accounted for by Malaysia (77% of participants). Therefore, the PAF estimates may be less robust for the Philippines. With increased follow-up of our cohort, more reliable estimates for the Philippines can be obtained as more events accrue. Finally, some risk factors had a high rate of missing values (particularly cholesterol and depression) (Supplemental Methods, Supplemental Table 4). Although we used a robust method of multiple imputation for the primary analysis, consistent with other PURE regional papers, future studies should aim to reduce rates of missing values to allow for more precise estimation.

Given the marked variations in risk factors, CVD, and mortality rates from 2 neighboring countries in Southeast Asia, our data emphasize the importance of obtaining prospective data from each of the several large countries in the region such as Indonesia, Vietnam, Thailand, and Myanmar.

## Conclusions

In Southeast Asia, CVD is the leading cause of all-cause mortality in both women and men. Hypertension is the leading risk factor for CVD, whereas low education is the leading risk factor for mortality (Central Illustration). Reducing CVD and mortality in adults will require a focus on improving educational attainment and the food system, as well as targeted measures to improve metabolic risk factors such as hypertension and diabetes.

Perspectives **COMPETENCY IN PATIENT CARE AND PROCEDURAL SKILLS:** The burden of CVD, all-cause mortality, and their determinants differs in various countries. Understanding these differences is essential in developing context-specific health policies. Prospective countrywide studies can document the incidence rates of CVD and all-cause mortality and can clarify the contribution of risk factors. A standardized cohort of participants from countries within Southeast Asia can provide necessary information on whether the rates of CVD and all-cause mortality vary, as well as region-specific data on their determinants.

**TRANSLATIONAL OUTLOOK:** In Southeast Asia, rates of CVD are higher than in the Philippines, whereas rates of all-cause mortality are higher in Malaysia, with important differences in risk factor profiles between the 2 countries. A focus on education will likely have the highest impact in reducing premature mortality in the region, whereas hypertension control should be the highest priority for reducing CVD. Our findings also underscore the importance of obtaining prospective countrywide data in the region, to inform health policies.

**Central Illustration:**
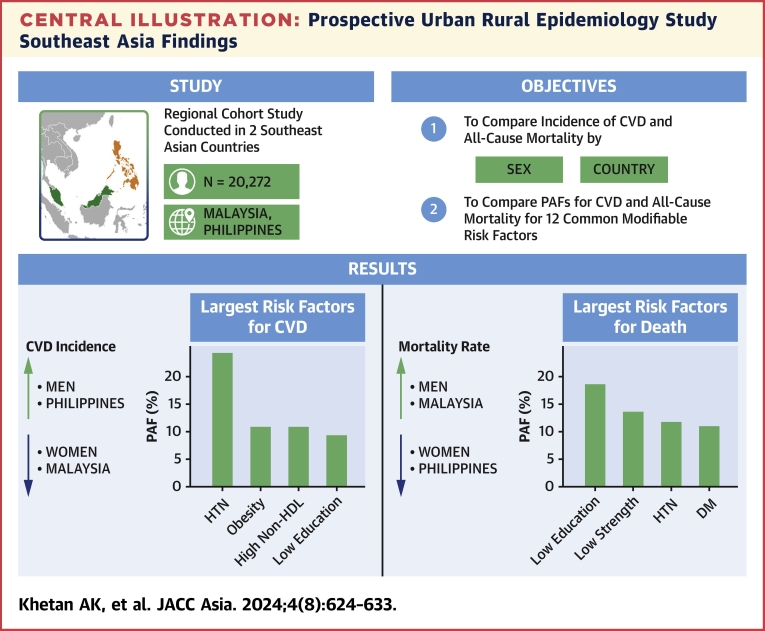
Prospective Urban Rural Epidemiology Study Southeast Asia Findings The incidence of cardiovascular disease (CVD) and all-cause mortality in Southeast Asia (Malaysia and the Philippines), as well as their key drivers. DM = diabetes mellitus; HDL = high-density lipoprotein; HTN = hypertension; PAF = population-attributable fraction.

## Funding Support and Author Disclosures

The PURE study is an investigator-initiated study that has received funding from the Population Health Research Institute, the Hamilton Health Sciences Research Institute, the Canadian Institutes of Health Research, the Heart and Stroke Foundation of Ontario, the Canadian Institutes of Health Research’s Strategy for Patient Oriented Research, the Ontario SPOR Support Unit, and the Ontario Ministry of Health and Long-Term Care; has received unrestricted grants from AstraZeneca (Canada), Sanofi Aventis (France and Canada), Boehringer Ingelheim (Germany and Canada), Servier, GlaxoSmithKline, Novartis, and King Pharma; and has received funding from various national or local organizations in participating countries: Argentina: Fundacion ECLA (Estudios Clínicos Latino America); Bangladesh: Independent University, Bangladesh, and Mitra and Associates; Brazil: Unilever Health Institute, Brazil; Canada: Public Health Agency of Canada and Champlain Cardiovascular Disease Prevention Network; Chile: Universidad de la Frontera; China: National Center for Cardiovascular Diseases and ThinkTank Research Center for Health Development; Colombia: Colciencias (grants 6566-04-18062 and 6517-777-58228); India: Indian Council of Medical Research; Malaysia: Ministry of Science, Technology and Innovation of Malaysia (grants 100-IRDC/BIOTEK 16/6/21 [13/2007] and 07-05-IFN-BPH 010), Ministry of Higher Education of Malaysia (grant 600-RMI/LRGS/5/3 [2/2011]), MARA Technological University, Kebangsaan University Malaysia (UKM-Hejim-Komuniti-15-2010); occupied Palestinian territory: the United Nations Relief and Works Agency for Palestine Refugees in the Near East, International Development Research Centre (Canada); Philippines: Philippine Council for Health Research and Development; Poland: Polish Ministry of Science and Higher Education (grant 290/W-PURE/2008/0), Wroclaw Medical University; Saudi Arabia: Saudi Heart Association, Saudi Gastroenterology Association, Dr Mohammad Alfagih Hospital, The Deanship of Scientific Research at King Saud University (research group RG -1436-013); South Africa: The North-West University, SA and Netherlands Programme for Alternative Development, National Research Foundation, Medical Research Council of South Africa, The South Africa Sugar Association, Faculty of Community and Health Sciences; Sweden: grants from the Swedish state under the Agreement Concerning Research and Education of Doctors, the Swedish Heart and Lung Foundation, the Swedish Research Council, the Swedish Council for Health, Working Life and Welfare, King Gustaf V’s and Queen Victoria Freemason’s Foundation, and AFA Insurance; Turkey: the Metabolic Syndrome Society, AstraZeneca, Sanofi Aventis; United Arab Emirates: Sheikh Hamdan Bin Rashid Al Maktoum Award for Medical Sciences and Dubai Health Authority. The external funders and sponsors had no role in the design and conduct of the study; in the collection, analysis, and interpretation of the data; in the preparation, review, or approval of the manuscript; or in the decision to submit the manuscript for publication. Dr Yusuf has received support from the Marion W. Burke endowed chair of the Heart and Stroke Foundation of Ontario. All other authors have reported that they have no relationships relevant to the contents of this paper to disclose.
