# Supplementary material for: Mortality, Cardiovascular Disease, and Their Associations With Risk Factors in Southeast Asia: A PURE Substudy
Source: JACC Asia. 2024 Jul 30;4(8):624–33. doi: 10.1016/j.jacasi.2024.05.008 (PMC11328768; doi:10.1016/j.jacasi.2024.05.008)
Supplement: Supplemental Material [file mmc1.docx]

**Supplemental Table 1. Definition of risk factors and thresholds for calculation of population attributable fractions**

| **Risk Factor** | **Definition or method of measurement** | **Risk category used for calculation of PAF** | **Reference category used for calculation of PAF** |
| --- | --- | --- | --- |
| **Behavioral cluster of risk factors:** | | | |
| Tobacco use | Self-reported tobacco consumption using a standard tobacco use frequency questionnaire, categorized as never, former or current. | History of current or former tobacco use | No history of tobacco use |
| Alcohol | Self-reported alcohol consumption using a standard alcohol consumption frequency questionnaire. Consumption was categorized as former, never and current. Current consumption is further categorized as low (<=7 drinks/week), moderate (8-14 drinks/week in women or 8-21 drinks/week in men), or high consumption (> 14 drinks/week in women or >21 drinks/week in men). | Excess alcohol use defined as either high current use or former use | No history of alcohol consumption, low current use, or moderate current use. |
| Diet | Diet was measured using country specific, food frequency dietary questionnaires (FFQ). Using these, a comprehensive diet score was created based on eight food types associated with a lower risk of CVD or mortality in PURE: fruits, vegetables, legumes, nuts, fish, dairy, unprocessed red meat and poultry; with each classified into high-consumption (1 point) or low-consumption (0 points) based on the median amount consumed in PURE (in grams/day), then added to a final score (with lower scores representing a lower quality diet) | Diet score < 4 | Diet score > 5 |
| Physical activity | Physical activity was measured using the International Physical Activity Questionnaire, and classified as low (<600 metabolic equivalents [MET] × minutes per week or <150 minutes per week of moderate intensity physical activity), moderate (600–3000 MET × minutes or 150–750 minutes per week) or high (>3000 MET × minutes or >750 minutes per week) | Low physical activity level | Moderate or high physical activity |
| **Metabolic cluster of risk factors:** | | | |
| Hypertension | Blood pressure was measured in all participants at baseline, and hypertension was defined as a systolic blood pressure > 140 mmHg, a self-reported history of hypertension, or treatment with anti-hypertensive medications. BP was measured by trained research assistants using a digital sphygmomanometer (Omron HEM-757; Omron Healthcare Co. Ltd., Scarborough, Ontario, Canada) with a cuff size of 14 􏰂 48 cm supplied to all sites [19,20]. Measurements were taken after the participant was seated for at least 5 min and not having smoked, exercised, eaten or climbed stairs in the last 30 min. BP was taken twice, with a 5-min interval between measurements, with the participant sitting upright and his/her right arm supported at heart level. The mean of the two measures was used in the analysis.  Hypertension was defined by self-report of the disease, taking BP-lowering treatment, or an average SBP at least 140 mmHg and/or an average DBP at least 90 mmHg measured during the visit | Definition of hypertension was met | Definition of hypertension was not met |
| Diabetes | Diabetes was defined as either a fasting glucose > 7 mmol/dl or self-reported history of diabetes, on treatment for diabetes. | Definition of diabetes was met | Definition of diabetes was not met |
| Non-HDL cholesterol | Total cholesterol minus HDL, measured using fasting lipid values | Highest two tertiles, corresponding to a value of >3.2 mmol/L | Lowest tertile of TC-HDL |
| Abdominal obesity | Waist and hip circumference were measured routinely in participants at baseline, and used to calculate the waist to hip ratio (WHR) | WHR > 0.9 in men or 0.85 in women | WHR < 0.9 in men or 0.85 in women |
| **Socio-economic and psychosocial cluster of risk factors:** | | | |
| Education | Education was self-reported, and classified as low (primary education level or less), intermediate (secondary school education) or high (college, trade school, or university education) | Low education | Intermediate or high education |
| Symptoms of Depression | Symptoms of depression were reported based on adapted version of the Short-Form Composite International Diagnostic Interview (CIDI-SF) for major depressive disorders, using an 8-point depression score based self-reported symptoms associated with depression. Symptoms consistent with depression was defined as a score > 5 | Symptoms consistent with depression | No depression |
| Grip strength | Measured using JAMAR dynamometer | Lowest two quintiles of grip strength | Highest three quintiles of grip strength |
| **Air pollution cluster of risk factors:** | | | |
| Household air pollution from kerosene or solid fuel use for cooking | Self-reported primary use of solid fuels (i.e. charcoal, coal, wood, agriculture/crop, animal dung, shrub/grass), kerosene, gas or electricity for cooking | Primary use of kerosene or solid fuel) | Primary use of gas or electricity for cooking |

Threshold of risk are based on our global PURE risk factor paper.^8^ This approach was decided because risk factor level differ in different regions of the world, and a standard threshold is necessary to allow for comparisons between regions and to the global data. For several risk factors where the associated risk is along a continuum, choosing extreme counterfactuals or reference values would inflate their impact by a modest degree, but would be difficult to achieve in any population-based strategy (i.e. shifting all individuals to a college/university of trade school education, or shifting all individuals to a high physical activity level). Therefore, we considered more conservative reference categories. For alcohol, we observed that high and former alcohol consumption were both associated with higher risk of mortality in our global paper, suggesting that in the former group, participants stopped alcohol consumption after suffering the adverse health effects of alcohol. Therefore, our risk group for calculation of the PAF related to alcohol combined the harmful effects from former and high alcohol consumption.

**Supplemental Table 2. Average Population Attributable Fraction (Average PAF, Mutually Adjusted) of Events due to a Group of Risk Factors: Additionally Adjusting for Age, Sex, and Location in South East Asia**

| **Exposure** | | | **Major CVD** | | **Mortality** | |
| --- | --- | --- | --- | --- | --- | --- |
| **Exposed Group** | **Reference** | **Percent Exposed** | **PAF(95% CI)** | **Rank** | **PAF(95% CI)** | **Rank** |
| Hypertension | No | 47.1 | 0.242 ( 0.088 , 0.396 ) | 1 | 0.122 ( 0.047 , 0.198 ) | 3 |
| Abdominal Obesity | No | 54.4 | 0.117 ( 0.037 , 0.198 ) | 2 | 0.018 ( -0.104 , 0.14 ) | 8 |
| High Non-HDL (>3.2 mmol/L) | Non-HDL <= 3.2 mmol/L | 84.3 | 0.116 ( -0.009 , 0.241 ) | 3 | 0.034 ( -0.102 , 0.17 ) | 7 |
| None or Primary Education | Secondary or Higher | 37.5 | 0.099 ( 0.025 , 0.173 ) | 4 | 0.184 ( 0.088 , 0.281 ) | 1 |
| Low Grip Strength (Q1 and Q2) | High (Q3-Q5) | 60.9 | 0.096 ( 0.035 , 0.158 ) | 5 | 0.134 ( 0.026 , 0.243 ) | 2 |
| Tobacco Use (Current + Former) | Never | 23.7 | 0.094 ( 0.039 , 0.149 ) | 6 | 0.097 ( 0.001 , 0.192 ) | 5 |
| Diabetes | No | 14.2 | 0.08 ( 0.017 , 0.142 ) | 7 | 0.112 ( 0.05 , 0.174 ) | 4 |
| Low or No Physical Activity | Moderate or High Activity | 27.2 | 0.018 ( -0.062 , 0.098 ) | 8 | 0.073 ( 0.027 , 0.118 ) | 6 |
| Depression Score >=4 | Score < 4 | 4.6 | -0.003 ( -0.022 , 0.016 ) | 9 | 0.011 ( -0.011 , 0.032 ) | 9 |
| Alcohol Use (Current High + Former) | Never/Low/Mod Drinker | 3.9 | -0.008 ( -0.046 , 0.031 ) | 10 | -0.014 ( -0.038 , 0.01 ) | 10 |
| Solid Fuel | Clean Fuel | 11.1 | -0.021 ( -0.107 , 0.064 ) | 11 | -0.062 ( -0.092 , -0.033 ) | 12 |
| Poor Diet (Score <=4) | Healthy Diet (Score>=5) | 69.6 | -0.055 ( -0.283 , 0.173 ) | 12 | -0.032 ( -0.156 , 0.092 ) | 11 |
| Total (All 12 Risk Factors) |  |  | 0.775 ( 0.659 , 0.891 ) | . | 0.677 ( 0.52 , 0.834 ) | . |
|  |  |  |  |  |  |  |
| Metabolic: Diab, Hyp, Obesity, Lipid |  |  | 0.555 | . | 0.286 | . |
| Unhealthy Behavior: UnH diet, Alcohol, Tobacco, Phys Inactive. |  |  | 0.049 | . | 0.124 | . |
| INTERHEART Modifiable Risk Factors + Education |  |  | 0.7 | . | 0.605 | . |

**Supplemental Table 3. Leading causes of death overall, by country and by sex.**

|  | Overall | Malaysia | Philippines | Women | Men |
| --- | --- | --- | --- | --- | --- |
| Total deaths | 2262 | 2049 | 213 | 936 | 1326 |
| CVD | 37.9 | 36.3 | 58.4 | 36.2 | 39.2 |
| Cancer | 12.4 | 12.3 | 13.4 | 15.9 | 9.9 |
| Injury | 4.4 | 4.5 | 2.0 | 1.9 | 6.1 |
| Respiratory | 10.5 | 10.6 | 8.7 | 8.8 | 11.6 |
| Infections | 8.7 | 9.2 | 2.7 | 9.3 | 8.3 |
| GI | 2.9 | 2.8 | 4.0 | 2.6 | 3.1 |
| Other causes | 6.9 | 7.2 | 3.4 | 9.3 | 5.3 |
| Unknown | 16.4 | 17.1 | 7.4 | 16.1 | 16.5 |

CVD, cardiovascular disease; GI, gastrointestinal

**Supplemental Table 4. Percent of missing values for each of the 12 risk factors evaluated in the study**

|  | % of missing data |
| --- | --- |
| Education | 0% |
| Tobacco Use | 0% |
| Diabetes | 0% |
| Physical Activity | 5% |
| Abdominal Obesity | 14% |
| Hypertension | 18% |
| Diet | 20% |
| Grip Strength | 25% |
| Dirty Cooking Fuel | 25% |
| Non HDL Cholesterol | 47% |
| Depression | 88% |

**Supplemental Methods**

**PURE Study Participant Selection Methodology as Excerpted from Teo et al. Am Heart J. 2009 Jul; 158 (1):1-7**

**Selection of Communities**

Within each country, urban and rural communities were selected based on broad guidelines (see Guidelines for Selection of Countries, Communities, Households, and Individuals Recruited to PURE). A common definition for “community” that is applicable globally is difficult to establish. In PURE, a community was defined as a group of people who have common characteristics and reside in a defined geographic area. A city or large town was not usually considered a single community, rather communities from low-, middle-, and high-income areas were selected from sections of the city and the community area defined according to a geographical measure (e.g., a set of contiguous postal code areas or a group of streets or a village). The primary sampling unit for rural areas in many countries was the village. The reason for inclusion of both urban and rural communities is that for many countries, urban and rural environments exhibit distinct characteristics in social and physical environment, and hence, by sampling both, we ensured considerable variation in societal factors across PURE communities.

The number of communities selected in each country varied, with the aim to recruit communities with substantial heterogeneity in social and economic circumstances balanced against the capacity of local investigators to maintain follow-up. In some countries (e.g., India, China, Canada, and Colombia), communities from several states/provinces were included to capture regional diversity, in policy, socioeconomic status, culture, and physical environment.

**Selections of Households and Individuals**

Within each community, sampling was designed to achieve a broadly representative sample of that community of adults aged between 35 and 70 years (see Table below for general recommendations for countries in PURE). The choice of sampling frame within each center was based on both “representativeness” and feasibility of long-term follow-up, following broad study guidelines. Once a community was identified, where possible, common and standardized approaches were applied to the enumeration of households, identification of individuals, recruitment procedures, and data collection.

The method of approaching households differed between regions. For example, in rural areas of India and China, a community announcement was made to the village through contact of a community leader, followed by in-person door-to-door visits of all households.

Households were eligible if at least 1 member of the household was between the ages of 35 and 70 years and the household members intended to continue living in their current home for a further 4 years.

For each approach, at least 3 attempts at contact were made. All individuals within these households between 35 and 70 years providing written informed consent were enrolled. When an eligible household or eligible individual in a household refused to participate, demographics and self-reported data about CVD risk factors, education, and history of CVD, cancers and deaths in the households within the 2 previous years were recorded.

To ensure standardization and high data quality, we used a comprehensive operations manual, training workshops, DVDs, regular communication with study personnel and standardized report forms. We entered all data in a customized database programmed with range and consistency checks, which was transmitted, electronically to the Population Health Research Institute in Hamilton (Ontario, Canada) where further quality checks were implemented.

**Guidelines for Selection of Countries, Communities, Households, and Individuals Recruited to PURE**

| **Countries** |
| --- |
| 1. High-income countries, middle-income countries, and low-income countries, with the bulk of the recruitment from low- and middle-income regions. |
| 2. Committed local investigators with experience in recruiting for population studies. |
| **Communities** |
| 1. Select both urban and rural communities. Use the national definition of the country to determine urban and rural communities. |
| 2. Select rural communities that are isolated (distance of >50 km or lack easy access to commuter transportation) from urban centers. However, consider ability to process bloods samples, e.g., villages in rural developing countries should be within 45-min drive of an appropriate facility. |
| 3. Define community to a geographical area, e.g., using postal codes, catchment area of health service/clinics, census tracts, areas bordered by specific streets or natural borders such as a river bank. |
| 4. Consider feasibility for long-term follow-up, e.g., for urban communities, choose sites that have a stable population such as residential colonies related to specific work sites in developing countries. In rural areas, choose villages that have a stable population. Villages at greater distance from urban centers are less susceptible to large migration to urban centers. |
| 5. Enlist a community organization to facilitate contact with the community, eg, in urban areas, large employers (government and private), insurance companies, clubs, religious organizations, clinic or hospital service regions. In rural areas, local authorities such as priests or community elders, hospital or clinic, village leader, or local politician. |
| **Individual** |
| 1. Broadly representative sampling of adults 35 to 70 years within each community unit. |
| 2. Consider feasibility for long-term follow-up when formulating community sampling framework, e.g., small percentage random samples of large communities may be more difficult to follow-up because they are dispersed by distance. In rural areas of developing countries that are not connected by telephone, it may be better to sample entire community (i.e., door-to-door systematic sampling). |
| 3. The method of approach of households/individuals may differ between sites. In MIC and HIC, mail, followed up by phone contact may be the practical first means of contact. In LIC, direct household contact through household visits may be the most appropriate means of first contact. |
| 4. Once recruited, all individuals are invited to a study clinic to complete standardized questionnaires and have a standardized set of measurements. |

**Standardized Event Definitions in PURE as excerpted from Yusuf et al. Lancet. 2020 Mar 7;395(10226):795-808**

**Prospective follow-up for cardiovascular events and death:** History of disease was collected at baseline from every participant with standardized questionnaires regarding history of a) hypertension, b) diabetes c) stroke d) angina/myocardial infarction/coronary artery disease e) heart failure f) other heart disease.

Information on specific events (death, myocardial infarction, stroke, heart failure, cancer, hospitalizations, new diabetes, injury, tuberculosis, human immunodeficiency viral infections, malaria, pneumonia, asthma, chronic obstructive pulmonary disease) were obtained from participants or their family members (events were reported by the participants if alive or by a relative if the individual had died). This information was adjudicated centrally in each country by trained physicians using standardized definitions. Because the PURE study involves urban and rural areas from middle- and low-income countries, supporting documents to confirm cause of death and/or event varied in degrees of completion and availability. In most of middle- and low-income countries there was no central system of death or event registration. Therefore, information was obtained about prior medical illness and medically certified cause of death where available, and, second, best available information was captured from reliable sources in those instances where medical information was not available in order to be able to arrive at a probable diagnosis or cause of death. Event documentation was based on information from household interviews and medical records, death certificates and other sources. Verbal autopsies were also used to ascertain cause of death in addition to medical records which were reviewed by a health professional. This approach has been used in several studies conducted in middle- and low-income countries.

To ensure a standard approach and accuracy for classification of events across all countries and over time, the first 100 CVD events (deaths, MI, strokes, heart failure or cancers) for China and India, and 50 cases for other countries were adjudicated both locally and also by the adjudication chair, and if necessary further training was provided. Thereafter, every year, 50 cases for China and India and 25 cases for each of the remaining countries were adjudicated as above.

**CVD Definitions and Coding**

FATAL EVENTS

Cardiovascular Death – Definitions

01.00 DEATH DUE TO CARDIOVASCULAR EVENTS

01.10 Sudden unexpected Cardiovascular Death (SCVD)

Without evidence of other cause of death, death that occurred suddenly and unexpectedly (examples: witnessed collapse, persons resuscitated from cardiac arrest who later died) or persons seen alive less than 12 hours prior to discovery of death (example persons found dead in his/her bed).

01.10 SCVD is either definite, probable or possible according to the following characteristics:

| PURE  Adjudication Code | Event Type | Acceptable ICD-10 codes |
| --- | --- | --- |
| 01.11: Definite | One of the following in persons with:   - known cardiovascular disease, or - diabetes with an additional risk factor such as hypertension, smoking, dyslipidemia, micro albuminuria, serum creatinine 50% above upper limit of normal, or - 3 of the above risk factors, or - 2 of the above risk factors in men aged 60 and more and women aged 65 and more | No ICD-10 Code |
| 01.12: Probable | One of the following in persons with:   - diabetes, or - 2 of the above risk factors in men aged less than 60 and in women less than 65, or - one of the above risk factor in men aged 60 and more and in women aged 65 and more, or - typical of chest pain or sudden severe dyspnea of less than 20-minute duration preceding the event |  |
| 01.13: Possible | In persons without risk factor |  |
| *For SCVD, the patient was well or had a stable CVD (example stable angina) when last seen alive. The event of a sudden death occurring during the hospitalization of MI is considered a fatal MI and not sudden death.* | | |

01.3 Fatal Myocardial Infarction (MI)

Symptoms of Myocardial Infarction:

Typical symptoms or suggestive symptoms of MI according to physician are characterized by severe anterior chest pain as tightness, crushing, burning, lasting at least 20 minutes, occurring at rest, or on exertion, that may radiate to the arms or neck or jaw and may be associated with dyspnea, diaphoresis and nausea. However, death associated with nausea and vomiting with or without chest pain not due to another cause may be considered as possible MI if ECG and cardiac markers are not done. These symptoms may have occurred the last month before death.

Fatal myocardial infarction is either definite, probable or possible according to the following characteristics:

| PURE  Adjudication Code | Event Type | Acceptable ICD-10 codes |
| --- | --- | --- |
| 01.31: Definite | 1. Autopsy demonstrating fresh myocardial infarction and/or recent coronary occlusion, or 2. ECG showing new and definite sign of MI (Minnesota code 1-1-1) or 3. Symptoms typical or atypical or inadequately described but attributed to cardiac origin lasting at least 20 minutes and by troponin or cardiac enzymes (CKMB, CK, SGOT, SLDH) above center laboratory ULN 4. ECG with new ischemic changes (new ST elevation/depression or T wave inversion ≥ 2 mm) and by troponin or cardiac enzymes (CKMB, CK, SGOT, SLDH) above center laboratory ULN | I21- I22 |
| 01.32: Probable | 1. ECG with sign of probable MI (Minnesota code 1-2-1), or 2. Typical symptoms lasting at least 20 minutes considered of cardiac origin, with only new ST-T changes (new ST elevation/depression or T wave inversion ≥ 1 but < 2mm) without documented increased cardiac markers or enzyme as in PURE definition 1.31 (above), or 3. Increased cardiac enzymes as in PURE definition 1.31 (above) showing a typical pattern of MI as above without symptoms or significant ECG changes |  |
| 01.33: Possible | 1. ECG with sign of possible MI (Minnesota code 1-3-1) or 2. Typical symptoms or symptoms suggestive of MI according to the physician lasting at least 20 minutes without documented ECG or cardiac marker. |  |

The Minnesota codes for MI is taken from Rose and Blackburn and published in their book “Evaluation Methods of Cardiovascular Disease WHO 1969”.

- Definite  MI is Q/R ratio ≥1/3 and Q duration ≥ 0.03 second in one of the following leads: I, II, V2, 3, 4, 5, 6. (code 1-1-1)
- Probable MI is Q/R ratio ≥1/3 and Q duration between 0.02 and  0.03 second in one of the following leads: I, II, V2, 3, 4, 5, 6. (code 1-2-1)
- Possible  MI is Q/R ratio between 1/5 and 1/3 and Q duration between 0.02 and  0.03 second in one of the following leads: I, II, V2, 3, 4, 5, 6. (code 1-3-1)

01.40 Fatal Stroke

Fatal stroke is either definite or possible according to the following characteristics:

| PURE  Adjudication Code | Event Type | Acceptable ICD-10 codes |
| --- | --- | --- |
| 01.41: Definite | Stroke death is defined as death within 30 days from an acute focal neurological deficit *diagnosed by a physician* and thought to be of vascular origin (without other cause such as brain tumor) with signs and symptoms lasting >= 24 hrs.  Stroke death is also considered if death occurred within 24 hrs. of onset of persisting signs and symptoms, or if there is evidence of a recent stroke on autopsy.  N.B.   - In a subject with a stroke <= 30 days: If death occurred with a pneumonia due to possible aspiration, death will be considered to be due to stroke. - In a subject with a stroke > 30 days: If death occurred with a pneumonia due to possible aspiration, the adjudicator will make a decision according to his/her clinical judgment if death is related to stroke or not. - Subarachnoid hemorrhage death manifested by sudden onset headache with/without focal signs and imaging (CT or MRI) evidence of bleeding primarily in the subarachnoid space is considered a fatal stroke in absence of trauma or brain tumor or malformation - Subdural hematoma death is not considered as a stroke death and may be related to previous trauma or other cause. | I60- I64, I69 |
| 01.43: Possible | Death in a participant with a history of sudden onset of focal neurological deficit of one or more limbs, loss of vision or slurred speech lasting about 24 hours. |  |

01.50 Fatal Congestive Heart Failure

Fatal congestive heart failure is either definite or possible according to the following characteristics:

| PURE  Adjudication Code | Event Type | Acceptable ICD-10 codes |
| --- | --- | --- |
| 01.51: Definite | The diagnosis of congestive heart failure may be an autopsy finding in absence of other cause or requires signs (rales, increased jugular venous pressure or ankle edema) or symptoms (nocturnal paroxysmal dyspnea, dyspnea at rest or ankle edema) of congestive heart failure and one or both of the following:   - radiological signs of pulmonary congestion, - treatment of heart failure with diuretics   *If sudden death occurred in a patient with chronic severe heart failure, it should be adjudicated as fatal congestive heart failure.* | I50 |
| 01.52: Probable | Progressive shortness of breath on lying down or at night, improving on sitting up AND any of the following signs or symptoms: swelling of feet, distension of abdomen, progressive cough in a person with known hypertension or a history of previous MI/angina or other heart disease |  |
| 01.53: Possible | Progressive shortness of breath on lying down or at night, improving on sitting up AND any of the following signs or symptoms: swelling of feet, distension of abdomen, progressive cough |  |

01.60 Death Due to Other Cardiovascular Deaths *(other causes [1.10 to 1.50 above] having been excluded)*

| PURE  Adjudication Code | Event Type | Acceptable ICD-10 codes |
| --- | --- | --- |
| 01.61 | Arterial rupture of aneurysm | I71- I72 |
| 01.62 | Pulmonary embolism  *NOTE: Death associated with pulmonary embolism occurring within 2 weeks after a fracture such as hip, femur should attributed to death due to injury. Refer to Injury, Section 6.0* | I26 |
| 01.63 | Arrhythmic death (A-V block, sustained ventricular tachycardia in absence of other causes) | I44- I45,  I47- I49 |
| 01.64 | Death after invasive cardiovascular intervention: a perioperative death extending to 30 days after coronary or arterial surgical revascularization and to 7 days after a coronary or arterial percutaneous dilatation (angioplasty) with or without a stent or an invasive diagnostic procedure. | I97 |
| 01.65 | Congenital heart disease | Q20-Q28 |
| 01.66 | Heart valve disease (including rheumatic heart disease) | I01, I05- I09,  I34- I37 |
| 01.67 | Endocarditis | I33, I38 |
| 01.68 | Myocarditis | I40 |
| 01.69 | Tamponade (pericarditis) | I30, I31, I32 |
| 01.70 | Other cardiovascular events *(Excluding 1.61 to 1.69 above)*  *Valid ICD-10 codes would include the following:*  *I11, I12, I13, I23, I24, I25, I27, I28, I42, I51, I52, I65-I68, I73, I74, I96, I98, I99 (Refer to ICD-10 Listing for associated definitions for each code)* | Any valid ‘I’ (Cardiovascular) ICD-10 code that can be classified as underlying cause of death, not specified above |

NON-FATAL EVENTS

Cardiovascular Events – Definitions

10.00 NON-FATAL CARDIOVASCULAR EVENTS

10.10 Non-Periprocedural Myocardial Infarction (MI)

MI is considered either definite, probable or possible according to the following characteristics:

| PURE  Adjudication Code | Event Type | Acceptable ICD-10 codes |
| --- | --- | --- |
| 10.11: Definite | 1. ECG showing new and definite sign of MI (Minnesota code 1-1-1) or 2. Symptoms typical or atypical or inadequately described but attributed to cardiac origin lasting at least 20 minutes and by troponin or cardiac enzymes (CKMB, CK, SGOT, SLDH) above center laboratory ULN 3. ECG with new ischemic changes (new ST elevation/depression or T wave inversion ≥ 2 mm) and by troponin or cardiac enzymes (CKMB, CK, SGOT, SLDH) above center laboratory ULN   Please note that increased markers may occur in trauma (CK, AST, myoglobin and CK MB to a lesser degree); renal insufficiency, heart failure, pulmonary embolism (troponin), cardioversion (all) | I21-I22 |
| 10.12: Probable | 1. ECG with new and probable sign of MI (Minnesota code 1-2-1), or 2. Typical symptoms lasting at least 20 minutes considered of cardiac origin, with only new ST-T changes (new ST elevation/depression or T wave inversion ≥ 1 but < 2mm) without documented increased cardiac markers as in PURE definition 10.11 (above), or 3. Increased cardiac enzymes showing a typical pattern of MI as above without symptoms or significant ECG changes. |  |
| 10.13: Possible | 1. ECG with new and possible sign of MI (Minnesota code 1-3-1), or 2. Typical symptoms lasting 20 minutes and more considered to be of cardiac origin without documented ECG or cardiac marker. |  |

10.20 Periprocedural Myocardial Infarction

| PURE  Adjudication Code | Event Type | Acceptable ICD-10 codes |
| --- | --- | --- |
| 10.21: Definite | 1. ECG showing new and definite sign of MI (Minnesota code 1-1-1), or 2. Increased cardiac markers within 48 hours of procedure:  - percutaneous coronary intervention: CKMB should be ≥ 5 X ULN or troponin ≥ 5 X above lower level of necrosis OR > 20% increase in cardiac markers if elevated at the beginning of the procedure in a patient with symptoms suggestive of myocardial ischemia - Coronary surgery: Increased cardiac markers CKMB should be ≥ 10X ULN or troponin ≥ 10X above lower limit of necrosis. | I21-I22 |

The Minnesota codes for MI is taken from Rose and Blackburn and published in their book “Evaluation Methods of Cardiovascular Disease WHO 1969”.

- Definite  MI is Q/R ratio ≥1/3 and Q duration ≥ 0.03 second in one of the following leads: I, II, V2, 3, 4, 5, 6. (code 1-1-1)
- Probable MI is Q/R ratio ≥1/3 and Q duration between 0.02 and  0.03 second in one of the following leads: I, II, V2, 3, 4, 5, 6. (code 1-2-1)
- Possible  MI is Q/R ratio between 1/5 and 1/3 and Q duration between 0.02 and  0.03 second in one of the following leads: I, II, V2, 3, 4, 5, 6. (code 1-3-1)

10.30 Stroke/Transient Ischemic Attack (TIA)

| PURE  Adjudication Code | Event Type | Acceptable ICD-10 codes |
| --- | --- | --- |
| 10.31: Definite | Stroke is defined as an acute focal neurological deficit *diagnosed by a physician* and thought to be of vascular origin (without other case such as brain tumor) with signs and symptoms lasting ≥ 24 hrs.  N.B.   - Subarachnoid hemorrhage manifested by sudden onset headache with/without focal signs and imaging (CT or MRI or lumbar puncture) showing evidence of bleeding primarily in the subarachnoid space is considered a stroke in absence of trauma or brain tumor or malformation - Subdural hematoma is not considered as a stroke and may be related to previous trauma or other cause. | I60-I64, I69 |
| 10.33: Possible | Stroke is possible if there is a history of sudden onset of focal neurological deficit of one or more limbs, loss of vision or slurred speech lasting about 24 hours or more |  |
| 10.34: TIA | The diagnosis of TIA requires the presence of acute focal neurological deficit thought to be of vascular origin with signs and symptoms lasting less than 24 hours | G45 |

10.40 Congestive Heart Failure

| PURE  Adjudication Code | Event Type | Acceptable ICD-10 codes |
| --- | --- | --- |
| 10.41: Definite | The diagnosis of congestive heart failure requires signs (rales, increased jugular venous pressure or ankle edema) or symptoms (nocturnal paroxysmal dyspnea, dyspnea at rest or ankle edema) of congestive heart failure and one or both of the following:   - radiological signs of pulmonary congestion, - Treatment of heart failure with diuretics. | I50 |
| 10.42: Probable | Progressive shortness of breath on lying down or at night, improving on sitting up AND any of the following signs or symptoms: swelling of feet, distension of abdomen, progressive cough in a person with known hypertension or a history of previous MI/angina or other heart disease |  |
| 10.43: Possible | Congestive heart failure is considered possible when there is progressive shortness of breath on lying down or at night, improving on sitting up AND any of the following signs or symptoms: swelling of feet, distension of abdomen, progressive cough |  |

**Coding of Deaths in the PURE Study**

| **Generic Event Codes** | **Description** | **Acceptable FATAL PURE Codes** | | | | **Acceptable ICD 10 Code Ranges** |
| --- | --- | --- | --- | --- | --- | --- |
|  |  | **Definite** | **Probable** | **Possible** | **Codes for which Definite/**  **Probable/**  **Possible not defined** |  |
| **1.00** | **Death Due to Cardiovascular Events** |  |  |  |  |  |
| 1.10 | **Sudden unexpected cardiovascular death** | 1.11 | 1.12 | 1.13 |  | **No ICD Code** |
| 1.30 | **Fatal Myocardial Infarction** | 1.31 | 1.32 | 1.33 |  | **I21-I22** |
| 1.40 | **Fatal Stroke** | 1.41 |  | 1.43 |  | **I60-I64, I69** |
| 1.50 | **Fatal Congestive Heart Failure** | 1.51 | 1.52 | 1.53 |  | **I50** |
| 1.60 | **Other cardiovascular deaths** |  |  |  |  |  |
| - | **-** Arterial rupture of aneurysm |  |  |  | 1.61 | **I71-I72** |
| - | **-** Pulmonary Embolism |  |  |  | 1.62 | **I26** |
| - | **-** Arrhythmic death (A-V block, sustained ventricular tachycardia in absense of other causes) |  |  |  | 1.63 | **I44-I45, I47-I49** |
| - | **-** Death after invasive cardiovascular intervention |  |  |  | 1.64 | **I97** |
| - | **-** Congenital heart disease |  |  |  | 1.65 | **Q20-Q28** |
| - | **-** Heart valve disease (including RHD) |  |  |  | 1.66 | **I01, I05-I09, I34-I37** |
| - | **-** Endocarditis |  |  |  | 1.67 | **I33, I38, I39** |
| - | **-** Myocarditis |  |  |  | 1.68 | **I40, I41** |
| - | **-** Tamponade (Pericarditis) |  |  |  | 1.69 | **I30-I32** |
| - | **-** Other cardiovascular events |  |  |  | 1.70 | **I00, I02, I10-I13, I15, I23-I25, I27, I28, I42, I43, I51, I52, I65-I68, I70, I73, I74, I77-I83, I85-I89, I95, I98, I99** |
| **2.00** | **Death due to most frequent infections** |  |  |  |  |  |
| 2.10 | **Typhoid and Paratyphoid** | 2.11 | 2.12 |  |  | **A01** |
| 2.20 | **Diarrhoea and Gastroenteritis/Dysentery** | 2.21 | 2.22 |  |  | **A00, A02-A09** |
| 2.30 | **Pulmonary Tuberculosis** | 2.31 | 2.32 | 2.33 |  | **A15-A16, A19** |
| - | **Septicaemia** |  |  |  | 2.40 | **A40-A41** |
| 2.50 | **Viral Hepatitis** | 2.51 | 2.52 |  |  | **B15-B19** |
| 2.60 | **AIDS** | 2.61 | 2.62 |  |  | **B20-B24** |
| 2.70 | **Malaria** | 2.71 | 2.72 |  |  | **B50-B54** |
| 2.80 | **Covid-19** | 2.81 | 2.82 |  |  | **U07** |
| - | **Other Infections** |  |  |  | 2.90 | **A17-A18, A20-A39, A42-A99, B00-B09, B25-B49, B55-B99** |
| **3.00** | **Death due to cancer** |  |  |  |  |  |
| - | **Mouth** |  |  |  | 3.01 | **C00-C06, C10, C12, C14** |
| - | **Esophagus** |  |  |  | 3.02 | **C15** |
| - | **Stomach** |  |  |  | 3.03 | **C16** |
| - | **Small Intestine** |  |  |  | 3.04 | **C17** |
| - | **Large intestine including rectum** |  |  |  | 3.05 | **C18-C20** |
| - | **Pancreas** |  |  |  | 3.06 | **C25** |
| - | **Liver** |  |  |  | 3.07 | **C22, C24** |
| - | **Lung/Pleura** |  |  |  | 3.08 | **C33-C34, C38-C39, C45** |
| - | **Breast** |  |  |  | 3.09 | **C50** |
| - | **Prostate** |  |  |  | 3.11 | **C61** |
| - | **Head and Neck** |  |  |  | 3.13 | **C31-C32, C70** |
| - | **Skin** |  |  |  | 3.14 | **C43-C44** |
| - | **Multi-site** |  |  |  | 3.15 | **C97** |
| - | **Other, specify** |  |  |  | 3.16 | **C07-C09, C11, C13, C23, C26, C30, C37, C46-C49, C57-C58, C63, C68-C69, C74-C79, C96, D37-D45, D47, D48** |
| - | **Cervical** |  |  |  | 3.17 | **C53** |
| - | **Uterine/Ovarian** |  |  |  | 3.18 | **C54-C56** |
| - | **Vaginal/vulva** |  |  |  | 3.19 | **C51-C52** |
| - | **Kidney** |  |  |  | 3.20 | **C64, C65** |
| - | **Bladder** |  |  |  | 3.21 | **C66-C67** |
| - | **Anus** |  |  |  | 3.22 | **C21** |
| - | **Testis** |  |  |  | 3.23 | **C62** |
| - | **Penis** |  |  |  | 3.24 | **C60** |
| - | **Brain/spinal cord** |  |  |  | 3.25 | **C71-C72** |
| - | **Leukemia** |  |  |  | 3.26 | **C91-C95, D46** |
| - | **Lymphoma** |  |  |  | 3.27 | **C81-C86, C88** |
| - | **Multiple Myeloma** |  |  |  | 3.28 | **C90** |
| - | **Musculoskeletal (muscle, bones, tendons, ligaments, joints, cartilage)** |  |  |  | 3.29 | **C40-C41** |
| - | **Thyroid** |  |  |  | 3.30 | **C73** |
| - | **Unknown site** |  |  |  | 3.40 | **C80** |
| **4.00** | **4.00 Death due to diseases of the respiratory system** |  |  |  |  |  |
| 4.10 | **Pneumonia** | 4.11 | 4.12 | 4.13 |  | **J12-J18** |
| 4.30 | **Asthma** | 4.31 | 4.32 | 4.33 |  | **J45-J46** |
| 4.40 | **COPD (Obstructive airways disease)** | 4.41 | 4.42 | 4.43 |  | **J44** |
| - | **Other respiratory diseases** |  |  |  | 4.90 | **J00-J11, J20-J43, J47-J99, Q30-Q34** |
| **5.00** | **Death related to pregnancy/delivery/puerperium** |  |  |  |  |  |
| - | **Death related to pregnancy/delivery/ puerperium (Direct obstetrical causes)** |  |  |  | 5.00 | **O00-O99** |
| **6.00** | **Death due to Injury** |  |  |  |  |  |
| - | **Death due to Injury** |  |  |  | 6.00 | **V01-V99, W00-W99, X00-X99, Y00-Y98** |
| **7.00** | **Death due to other causes** |  |  |  |  |  |
| - | **Diseases of Nervous system** |  |  |  | 7.10 | **G00-G99, Q00-Q07** |
| - | **Diseases of Digestive System** |  |  |  | 7.20 | **K00-K93, Q39-Q45** |
| - | **Diseases of genito-urinary system** |  |  |  | 7.30 | **N00-N99, Q50-Q64** |
| - | **Other diseases, specify** |  |  |  | 7.50 | **D50-D89, E00-E90, F00-F99, H58, H59, H94, H95, L00-L99, M00-M99, Q35-Q38, Q65-Q99** |
| **8.00** | **Death due to unspecified/presumed cause** |  |  |  |  |  |
| - | **Presumed cardiovascular death** |  |  |  | 8.10 | **No ICD Code** |
| - | **Presumed cancer death** |  |  |  | 8.20 | **No ICD Code** |
| - | **Unknown death** |  |  |  | 8.30 | **R99** |

**Funding Support, PURE Investigators and Primary Country Based Institutions**

**Funding/Support:**

Dr. S. Yusuf is supported by the Marion W Burke endowed chair of the Heart and Stroke Foundation of Ontario.

The PURE study is an investigator-initiated study that is funded by the Population Health Research Institute, Hamilton Health Sciences Research Institute (HHSRI), the Canadian Institutes of Health Research, Heart and Stroke Foundation of Ontario, Support from Canadian Institutes of Health Research’s Strategy for Patient Oriented Research, through the Ontario SPOR Support Unit, as well as the Ontario Ministry of Health and Long-Term Care and through unrestricted grants from several pharmaceutical companies [with major contributions from AstraZeneca (Canada), Sanofi-Aventis (France and Canada), Boehringer Ingelheim (Germany and Canada), Servier, and GlaxoSmithKline], and additional contributions from Novartis and King Pharma and from various national or local organisations in participating countries.

These include: **Argentina:** Fundacion ECLA **(Estudios Clínicos Latino America)** ; **Bangladesh**: Independent University, Bangladesh and Mitra and Associates; **Brazil:** Unilever Health Institute, Brazil; **Canada:** Public Health Agency of Canada and Champlain Cardiovascular Disease Prevention Network; **Chile:** Universidad de la Frontera; **China:** National Center for Cardiovascular Diseases and ThinkTank Research Center for Health Development; **Colombia:** Colciencias (grant 6566-04-18062 and grant 6517-777-58228); **India:** Indian Council of Medical Research; **Malaysia:** Ministry of Science, Technology and Innovation of Malaysia (grant number: 100-IRDC/BIOTEK 16/6/21 [13/2007], and 07-05-IFN-BPH 010), Ministry of Higher Education of Malaysia (grant number: 600-RMI/LRGS/5/3 [2/2011]), Universiti Teknologi MARA, Universiti Kebangsaan Malaysia (UKM-Hejim-Komuniti-15-2010); **occupied Palestinian territory:** the United Nations Relief and Works Agency for Palestine Refugees in the Near East, occupied Palestinian territory; International Development Research Centre, Canada; **Philippines:** Philippine Council for Health Research and Development; **Poland:** Polish Ministry of Science and Higher Education (grant number: 290/W-PURE/2008/0), Wroclaw Medical University; **Saudi Arabia:** Saudi Heart Association, Saudi Gastroenterology Association, Dr.Mohammad Alfagih Hospital, The Deanship of Scientific Research at King Saud University, Riyadh, Saudi Arabia (Research group number: RG -1436-013); **South Africa:** The North-West University, SA and Netherlands Programme for Alternative Development, National Research Foundation, Medical Research Council of South Africa, The South Africa Sugar Association, Faculty of Community and Health Sciences; **Sweden:** Grants from the Swedish state under the Agreement concerning research and education of doctors; the Swedish Heart and Lung Foundation; the Swedish Research Council; the Swedish Council for Health, Working Life and Welfare, King Gustaf V:s and Queen Victoria Freemason’s Foundation, AFA Insurance; **Turkey:** Metabolic Syndrome Society, AstraZeneca, Sanofi Aventis; **United Arab Emirates:** Sheikh Hamdan Bin Rashid Al Maktoum Award For Medical Sciences and Dubai Health Authority, Dubai.

**Role of Sponsor:** The external funders and sponsors had no role in the design and conduct of the study; in the collection, analysis, and interpretation of the data; in the preparation, review, or approval of the

manuscript; or in the decision to submit the manuscript for publication.

**PURE Project Office Staff, National Coordinators, Investigators, and Key Staff:**

**Project office (Population Health Research Institute, Hamilton Health Sciences and McMaster University, Hamilton, Canada):** S Yusuf* (Principal Investigator).

S Rangarajan (Program Manager); K K Teo, S S Anand, C K Chow, M O’Donnell, A Mente, D Leong, A Smyth, P Joseph, M Duong, O Kurmi, R D’Souza, M Walli-Attaei, B Balaji, R Naito, S Islam (Statistician), W Hu (Statistician), C Ramasundarahettige (Statistician), P Sheridan (Statistician), S Bangdiwala, L Dyal, M Dehghan (Nutrition Epidemiologist), A Aliberti, A Reyes, A Zaki, B Connolly, B Zhang, D Agapay, D Krol, E McNeice, E Ramezani, F Shifaly, G McAlpine, I Kay, J Rimac, J Swallow, M Di Marino, M Jakymyshyn, M(a) Mushtaha, M(o) Mushtaha, M Trottier, N Aoucheva, N Kandy, P Mackie, R Buthool, R Patel, R Solano, S Gopal, S Ramacham, S Trottier

**Core Laboratories**: G Pare, M McQueen, S Lamers, J Keys (Hamilton), X Wang (Beijing, China), A Devanath (Bangalore, India).

**Argentina:** R Diaz*, A Orlandini, P Lamelas, M L Diaz, A Pascual, M Salvador, C Chacon; **Bangladesh:** O Rahman*, R Yusuf*, S A K S. Ahmed, T Choudhury, M Sintaha, A Khan, O Alam, N, Nayeem, S N Mitra, S Islam, F Pasha; **Brazil:** A Avezum*, C S Marcilio, A C Mattos, G B Oliveira; **Canada:**  K Teo***,** S Yusuf*****, Sumathy Rangarajan, A Arshad, B Bideri, I Kay, J Rimac, R Buthool, S Trottier, G Dagenais, P Poirier, G Turbide, AS Bourlaud, A LeBlanc De Bluts, M Cayer, I Tardif, M Pettigrew, S Lear, V de Jong, A N Saidy, V Kandola, E Corber, I Vukmirovich, D Gasevic, A Wielgosz, A Pipe, A Lefebvre, A Pepe, A Auclair, A Prémont, A S Bourlaud; **Chile:** F Lanas*, P Serón, M J Oliveros, F Cazor, Y Palacios; **China:** Li Wei*, Liu Lisheng*, Bo Jian, Hu Bo, Yin Lu, Zhao Wenhua, Zhang Hongye, Jia Xuan, Sun Yi, Wang Xingyu, Zhao Xiuwen, He Xinye, Chen Tao, Chen Hui, Chang Xiaohong, Deng Qing, Cheng Xiaoru, Deng Qing, Xie Liya, Liu Zhiguang, Li Juan, Li Jian, Liu Xu, Ren Bing, Sun Yi, Wang Wei, Wang Yang, Yang Jun, Zhai Yi, Zhang Hongye, Zhao Xiuwen,Zhu Manlu, Lu Fanghong, Wu Jianfang, Li Yindong, Hou Yan, Zhang Liangqing, Guo Baoxia, Liao Xiaoyang, Zhang Shiying, BianRongwen, TianXiuzhen, Li Dong, Chen Di, Wu Jianguo, Xiao Yize, Liu Tianlu, Zhang Peng, Dong Changlin, Li Ning, Ma Xiaolan, Yang Yuqing, Lei Rensheng, Fu Minfan, He Jing, Liu Yu, Xing Xiaojie, Zhou Qiang; **Colombia:** P Lopez-Jaramillo*, P A Camacho-Lopez, M Perez-Mayorga, J Otero-Wandurraga, JP Lopez-Lopez D I Molina, C Cure-Cure, JL Accini, E Hernandez, E Arcos, C Narvaez, A Sotomayor, F Manzur, H Garcia, G Sanchez, F Cotes, A Rico, M Duran, C Torres; **India: Bangalore -** P Mony *, M Vaz*, S Swaminathan, AV Bharathi, K Shankar, A V Kurpad, K G Jayachitra, H A L Hospital, AR Raju, S Niramala, V Hemalatha, K Murali, C Balaji, A Janaki, K Amaranadh, P Vijayalakshmi, **Chennai** - V Mohan*, R M Anjana, M Deepa, K Parthiban, L Dhanasekaran, SK Sundaram, M Rajalakshmi, P Rajaneesh, K Munusamy, M Anitha, S Hemavathy, T Rahulashankiruthiyayan, D Anitha, R. Dhanasekar, S. Sureshkumar, D Anitha, K Sridevi, **Jaipur** - R Gupta, R B Panwar, I Mohan, P Rastogi, S Rastogi, R Bhargava, M Sharma, D Sharma, **Trivandrum** - V Raman Kutty, K Vijayakumar, V Ambili, Arunlal AR Nair, K Ajayan, G Rajasree, AR Renjini, A Deepu, B Sandhya, S Asha, H S Soumya, **Chandigarh**- R Kumar, M Kaur, P V M Lakshmi, V Sagar J S Thakur, B Patro, R Mahajan, A Josh, G Singh, K Sharma, P Chaudary, **Iran:** R Kelishadi*, A Bahonar, N Mohammadifard, H Heidari, **Kazakhstan:** K Davletov*, B Assembekov, B Amirov; **Kyrgyzstan:** E Mirrakhimov*, S Abilova, U Zakirov, U Toktomamatov; **Malaysia: UiTM -** K Yusoff*, T S Ismail, K Ng, A Devi, N Mat-Nasir, AS Ramli, MNK Nor-Ashikin, R Dasiman, MY Mazaouspavina, F Ariffin, M Miskan, H Abul-Hamid, S Abdul-Razak, N Baharudin, NMN Mohd-Nasir, SF Badlishah-Sham, M Kaur, M Koshy, F A Majid, N A Bakar, N Zainon, R Salleh, SR Norlizan, NM Ghazali, M Baharom, H Zulkifli, R Razali, S Ali, CWJCW Hafar, F Basir; **UKM** - Noorhassim Ismail, M J Hasni, M T Azmi, M I Zaleha, R Ismail, K Y Hazdi, N Saian, A Jusoh, N Nasir, A Ayub, N Mohamed, A Jamaludin, Z Rahim; **Occupied Palestinian Territory:** R Khatib*, U Khammash, R Giacaman; **Pakistan:** R Iqbal*, R Khawaja, I Azam, K Kazmi; **Peru:** J Miranda*, A Bernabe Ortiz, W Checkley, R H Gilman, L Smeeth, R M Carrillo, M de los Angeles, C Tarazona Meza**;** **Philippines:** A Dans*, H U Co, J T Sanchez, L Pudol, C Zamora-Pudol, L A M Palileo-Villanueva, M R Aquino, C Abaquin, SL Pudol, K Manguiat, S Malayang; **Poland:** W Zatonski*, A Szuba, K Zatonska, R Ilow**^#^**, M Ferus, B Regulska-Ilow, D Różańska, M Wolyniec; **Saudi Arabia:** KF AlHabib*, M Alshamiri, HB Altaradi, O Alnobani, N Alkamel, M Ali, M Abdulrahman, R Nouri; **South Africa:** L Kruger^*^, A Kruger^#^, P Bestra, H Voster, A E Schutte, E Wentzel-Viljoen, FC Eloff, H de Ridder, H Moss, J Potgieter, A Roux, M Watson, G de Wet, A Olckers, J C Jerling, M Pieters, T Hoekstra, T Puoane, R Swart*, E Igumbor, L Tsolekile, K Ndayi, D Sanders, P Naidoo, N Steyn, N Peer, B Mayosi^#^, B Rayner, V Lambert, N Levitt, T Kolbe-Alexander, L Ntyintyane, G Hughes, J Fourie, M Muzigaba, S Xapa, N Gobile , K Ndayi, B Jwili, K Ndibaza, B Egbujie; **Sweden:** A Rosengren*, K Bengtsson Boström, A Rawshani, A Gustavsson, M Andreasson, L Wirdemann; **Tanzania:** K Yeates*, M Oresto, N West **Turkey:** A Oguz*, N Imeryuz, Y Altuntas, S Gulec, A Temizhan, K Karsidag, K B T Calik, A K Akalin, O T Caklili, M V Keskinler, K Yildiz; **United Arab Emirates:** A H Yusufali, F Hussain, M H S Abdelmotagali, D F Youssef, O Z S Ahmad, F H M Hashem, T M Mamdouh, F M AbdRabbou, S H Ahmed, M A AlOmairi, H M Swidan, M Omran, N A Monsef ; **Zimbabwe:** J Chifamba*, T Ncube, B Ncube, C Chimhete, G K Neya, T Manenji, L Gwaunza, V Mapara, G Terera, C Mahachi, P Murambiwa, R Mapanga, A Chinhara

*National Coordinator

^#^ Deceased

**Countries and Institution participating in PURE:**

|  | **Institution** |
| --- | --- |
| **South Africa** | Faculty of Health Science, North-West University, Potchefstroom Campus |
|  | University of the Western Cape, Department of Dietetics and Nutrition, Private Bag X17, 7535, Bellville, South Africa |
| **Zimbabwe** | University of Zimbabwe, College of Health Sciences, Physiology Department, Harare, Zimbabwe |
| **Tanzania** | Pamoja Tunaweza Women Center, Moshi, Tanzania  Division of Nephrology, Department of Medicine, Queen's University, Canada |
| **China** | National Centre for Cardiovascular Diseases, Cardiovascular Institute & Fuwai Hospital, Chinese Academy of Medical Sciences, 167, Bei Li Shi Lu, Beijing, China |
|  | Fuwai Hospital, 167 Beilishi Rd. Xicheng District, Beijing. 100037 China |
| **Philippines** | University of Philippines, Section of Adult Medicine & Medical Research Unit, Manila, Philippines |
| **Pakistan** | Department of Community Health Sciences and Medicine, Aga Khan University, Stadium Road, P.O Box 3500, Karachi Pakistan |
| **India** | St John's Medical College and Research Institute Bangalore 560034, India |
|  | Madras Diabetes Research Foundation &  Dr. Mohan’s Diabetes Specialities Centre, Chennai |
|  | Eternal Heart Care Centre and Research Institute, Jaipur |
|  | Health Action by People, Thiruvananthapuram, Kerala, 695011 INDIA |
|  | School of Public Health, Post Graduate Institute of Medical Education & Research, Chandigarh (India) |
| **Bangladesh** | Independent University, Bangladesh, Bashundhara, Dhaka, Bangladesh |
| **Malaysia** | Universiti Teknologi MARA, Sungai Buloh, Selangor, Malaysia AND UCSI University, Cheras, Selangor, Malaysia |
|  | Department of Community Health. Faculty of Medicine. University Kebangsaan Malaysia. Kuala Lumpur. Malaysia |
| **Poland** | Wroclaw Medical University Department of Internal Medicine; Department of Social Medicine Borowska 213 street; 50- 556 Wroclaw, Poland |
|  | Department of Epidemiology, The Maria Skłodowska-Curie Memorial Cancer Center and Institute of Oncology, 02-034 Warsaw, 15B Wawelska str. Poland |
| **Sweden** | Sahlgrenska Academy, University of Gothenburg, Sweden |
| **Russia** | Research Institute for Complex Issues of Cardiovascular Diseases, Kemerovo, Russia  Institute For Medical Education, Yaroslav-the-Wise Novgorod State University Ministry of Education and Science of the Russian Federation  Russia, Saint-Petersburg, 197022, Karpovka river emb., Bld.13, office 28 |
| **Turkey** | Istanbul Medeniyet University, Istanbul, Turkey |
| **Iran** | Isfahan Cardiovascular Research Center, Isfahan Research Institute  Isfahan University of Medical Sciences, Isfahan, Iran |
| **UAE** | Dubai Medical University, Hatta Hospital, Dubai Health Authority, Dubai, United Arab Emirates |
| **Saudi Arabia** | Department of Cardiac Sciences, King Fahad Cardiac Center  College of Medicine, King Saud University , Riyadh, Saudi Arabia |
| **Palestine** | Institute of Community and Public Health, Birzeit University, Ramallah, occupied Palestinian territory |
| **Kazakhstan** | Research Institute of Cardiology & Internal Diseases, Almaty, Kazakhstan |
| **Kyrgyzstan** | Kyrgyz Society of Cardiology, National Center of Cardiology and Internal Disease, Bishkek, Kyrgyzstan |
| **Canada** | Université Laval Institut universitaire de cardiologie et de pneumologie de Québec, Quebec, Canada G1V 4G5 |
|  | Simon Fraser University, Dept. of Biomedical Physiology & Kinesiology, BC, Canada |
|  | Department of Medicine, University of Ottawa, Ottawa, Canada |
|  | Population Health Research Institute, McMaster University, Hamilton Health Sciences, Hamilton, Ontario, Canada |
| **Argentina** | Estudios Clinicos Latinoamerica ECLA, Rosario, Santa Fe, Argentina  Department of Chronic Diseases, South American Center of Excellence for Cardiovascular Health (CESCAS)  Institute for Clinical Effectiveness and Health Policy (IECS) |
| **Brazil** | Dante Pazzanese Institute of Cardiology;  Hospital Alemao Oswaldo Cruz, Sao Paulo, SP Brazil |
| **Colombia** | Facultad de Ciencias de la Salud, Universidad de Santander (UDES), Bucaramanga, Santander, Fundacion Oftalmologica de Santander (FOSCAL), Floridablanca-Santander, Colombia |
| **Chile** | Universidad de La Frontera, Temuco, Chile |
| **Ecuador** | Facultad de Ciencias de la Salud Eugenio Espejo, Universidad Tecnológica Equinoccial, Dirección: Av. Mariscal Sucre s/n y Av. Mariana de Jesús, Quito Ecuador |
